# Symptom Burden and Dietary Changes Among Older Adults with Cancer: A Cross-Sectional Study

**DOI:** 10.3390/curroncol31120565

**Published:** 2024-12-01

**Authors:** Lea Büthe, Gina Westhofen, Andrea Hille, Judith Büntzel

**Affiliations:** 1 Department of Hematology and Medical Oncology, University Medical School, Robert-Koch-Straße 40, 37075 Göttingen, Germany; 2 Department of Radiation Therapy and Radiation Oncology, University Hospital Göttingen, 37075 Göttingen, Germany

**Keywords:** malnutrition, geriatric oncology, diet changes, symptom burden

## Abstract

Background: Malnutrition has a direct impact on both the toxicities of cancer therapy and the overall survival of oncological patients. However, its prevalence amongst vulnerable groups such as older patients (age ≥ 65 years) is often underestimated. Screening tools recognizing patients at risk are well established, yet they do not take into account that cancer therapy may lead to changes in dietary habits or that therapy’s side effects may negatively influence nutritional status. Methods: To close this gap, we combined the validated Nutritional Risk Score 2002 (NRS-2002) and G8 screening tools with short questionnaires addressing diet changes and symptom load and screened 300 cancer inpatients between 12/2022 and 12/2023. Descriptive statistics (Fisher’s exact, Student’s *t*-test) as well as heat mapping were applied for data analysis. Results: Overall, two in three inpatients ≥65 years were at risk for malnutrition, and the majority of patients (87.67%) scored ≤14 points on the G8 and were considered frail. Surprisingly, the symptom complex of oral discomfort was most often mentioned by patients (xerostomia—178/300 patients, loss of appetite: 122/300 patients, dysgeusia: 93/300 patients). Diet changes were also common, with patients mainly avoiding certain foods (122/300 patients) or using dietary supplements (106/300 patients). Conclusions: Taken together, older cancer inpatients are frail and have a high risk of malnutrition. Screening should not only consider energy intake but also symptom burden and dietary changes to optimize supportive care.

## 1. Introduction

Over 60% of all individuals diagnosed with cancer are aged 65 and over, and the prevalence of malnutrition amongst cancer patients is estimated to be 20–70% [1,2,3]. Guidelines define malnutrition either as a qualitative insufficiency due to the inadequate intake of nutrients and food components and/or as a quantitative (energetic) problem due to an insufficient caloric intake [2]. The underlying genesis of malnutrition is variable, as cancer itself but also treatment-related side effects might promote malnutrition. The transition from malnutrition toward cachexia or sarcopenia is possible and can occur at any stage of the disease, sometimes even before diagnosis [4]. Older people with cancer are a particularly vulnerable group due to being prone to lower caloric intake and following a less varied diet. Especially reduced food intake is often present before the onset of the disease [5]. The variability of the triggers for malnutrition is often underestimated, and guidelines emphasize considering patients’ symptom burden, as local impairments (e.g., dysphagia) as well as general symptoms affect nutritional status [6]. Battling malnutrition is essential. Malnourished patients have a worse prognosis [7]. Thus, recognizing these patients and addressing malnutrition directly affects the success of tumor therapy and patient survival. It is therefore of utmost importance to provide help and counseling as early as possible to counteract a negative spiral [8]. A range of screening instruments and assessments is available to uncover nutritional deficiencies. The selection of the appropriate tool depends on the screening setting and the disease entity or patient population [9]. In an inpatient setting, the Nutritional Risk Score-2002 (NRS-2002) [10] is often used as a screening tool, as recommended by guidelines [11]. The NRS-2002 is a tool combining a simple prescreen (yes/no questions) using questions about weight loss, decreased calorie intake, low body mass index (BMI), and severity of disease. If one question is answered with “yes”, patients undergo more detailed screening. Depending on the severity of disease or decrease in nutritional intake, points are assigned. A score ≥ 3 points considers a patient at risk for malnutrition [10]. However, the tool gives no information on symptoms leading to a changed nutrition intake. Further, a previous study has demonstrated that dietary changes are common in cancer patients and may influence nutritional status [12]. To address this information gap concerning both symptom load and dietary changes, we adapted our screening for patients at risk: based on the NRS-2002 [10] in combination with the G8 questionnaire [13], we extended our screening tool-box by recording symptom burden and dietary changes, as previously proposed [14]. We aimed to identify the older patients (age ≥ 65 years) at risk and the extent of symptom burden as well as dietary changes. Knowledge of patients’ diet and symptom load should then enable medical staff to optimize both nutrition and supportive cancer care.

## 2. Materials and Methods

*Patients and study design*. The study conducted is an anonymous cross-sectional study. Due to the exploratory character of the study, a timeframe for recruitment (one year) was predefined before starting the project. Data were collected prospectively between December 2022 and December 2023. A total of 300 patients treated as inpatients at the Department of Hematology and Medical Oncology at the University Medical Center Göttingen were included in the study and surveyed. Inclusion criteria were ≥65 years of age, an underlying oncological disease, an inpatient stay, and the ability to provide information as well as consent to participate. The aim was to offer participation to all inpatients who (1) fit the inclusion criteria and (2) were treated during the predefined recruitment time of one year. A yearlong screening was initiated to take into account seasonal fluctuation of patients. All patients were questioned by a previously trained member of the study group. The study’s protocol was approved by the Ethics Committee of the University of Göttingen (approval number 26/8/22). Patients were interviewed using an adapted screening tool. The components of this anamnesis questionnaire were the NRS-2002 [8], the Geriatric Screening G8 tool [13], a recently proposed questionnaire that considers dietary changes in cancer patients [14], and a question module on symptom burden. In addition, anthropometric data (age, sex, height, weight, BMI) and clinical data (initial diagnosis versus disease progression, tumor entity, and treatment modality) were collected. The NRS-2002 is suitable for screening patients with or at risk of malnutrition in an inpatient setting. It is recommended by the European Society for Clinical Nutrition and Metabolism for all hospitalized patients [4]. The Geriatric Screening G8 [13] is designed to identify patients who require more detailed geriatric assessment. A score ≤ 14 points is considered suspicious and should lead to reassessment or further investigation. Dietary changes and adherence to a cancer diet were assessed in a set of questions, as previously proposed by [12]. Patients were asked if they avoided or preferred certain foods, if they used dietary supplements, and if they followed a special or cancer diet. In addition to the established screening questionnaires, a symptom burden questionnaire was added. As part of this module, a list of symptoms that may be associated with malnutrition and oncological disease was discussed with the patients. Symptoms queried were as follows: oral mucositis, xerostomia, loss of appetite, dysgeusia, dysphagia, odynophagia, diarrhea, nausea, emesis, meteorism, abdominal discomfort, and constipation. Other symptoms could be listed in a free text box. The questionnaire (original German questionnaire, as well as a translated English version) is listed under Appendix A. The study has been reported in accordance with the STROBE guidelines [15], and the corresponding checklist is listed under Appendix B.

*Statistical analysis.* Due to smaller group sizes, some entities were summarized as follows: non-small lung cancer (NSCLC) and small-cell lung cancer (SCLC) as lung cancer; tumors of the cervix and vulva as other gynecological tumors; prostate, kidney, and urothelial tumors as urooncological tumors; and solid tumors of the jejunum, ileum, or colon as lower GI cancer. In addition, aggressive lymphoma and acute leukemia cases were grouped together, and chronic leukemia was grouped together with cases of indolent lymphoma, myelodysplastic (MDS), and myeloproliferative (MPN) neoplasms. Similarly, patients undergoing allogeneic or autologous stem cell therapy or CAR-T-cell therapy were considered participants receiving “cellular immunotherapy”. A treatment modality analysis was performed. A distinction was made between radiological therapy and systemic therapy. The subgroups were also analyzed with regard to different age cohorts. The following patient groups were analyzed: 65–74 years, 75–84 years, and ≥85 years. If data were missing for subgroup analysis, cases were excluded from analysis. The data were collected in Microsoft Excel. The statistical analysis was performed using Excel version 2021 and GraphPad Prism version 9.0. Descriptive statistics were applied to analyze demographic data. Fisher’s exact tests were used to test for differences. Generally, a *p*-value of *p* < 0.05 was considered statistically significant. A trend was defined as a *p*-value < 0.15. The following software was used to visualize data: GraphPad Prism (Version 9.3.0, GraphPad Software, Boston, MA, USA), Biorender [16], and wordcloud.com (accessed on on 28 September 2024) [17]. Heat-map analysis was conducted using the online freeware ClustVis [18].

## 3. Results

### 3.1. Risk of Malnutrition—An Ongoing Problem

Three hundred patients (136 female, 164 male) were included in this study. Hematological neoplasms were the most common diagnosis—187/300 patient cases (62.33%) versus 113/300 (37.67%) patients with solid tumors. Most patients with hematological neoplasms suffered from indolent (67/300, 22.33%) or aggressive lymphoma (45/300, 15%) and acute leukemia (39/300, 13.00%). Concerning solid tumors, lung cancer was the most common entity. A total of 25/300 (8.33%) patients received allogeneic, autologous stem cell therapy or CAR-T cell therapy. Further information on clinical baseline data is listed in Table 1.

At the time of inclusion, 43/300 (14.3%) patients were recently diagnosed with cancer. The average BMI was 24.99 kg/m^2^. There were no significant differences in the mean BMI between the age cohorts. A total of 239/300 (79.67%) of the hospitalized patients tested positive in the pre-screening using the NRS-2002, and a risk of malnutrition was confirmed in 209/300 (69.67%) in the main screening. Subgroup analysis was possible for the following groups: hematological neoplasm, solid tumors, aggressive lymphoma/acute leukemia, indolent lymphoma/chronic leukemia/MDS/MPN, lung cancer, and cellular immunotherapy. The malnutrition risk rates were as follows: 73.80% (138/187, hematological neoplasms), 62.83% (71/113, solid tumors), 73.81% (62/84, aggressive lymphoma/acute leukemia), 72.62% (61/84, indolent lymphoma/chronic leukemia/MDS/MPN), 61.36% (27/44, lung cancer), and 64.00% (16/25, cellular immunotherapy). Comparing different cancer entities, we observed a trend toward a higher risk for malnutrition in patients with hematological neoplasms (Table 2).

Furthermore, patients suffering from SCLC or undergoing cellular immunotherapy have a significantly lower risk of malnutrition.

To consider both radiation and systemic therapy, treatment modality was recorded separately after piloting. The distribution of patient cases was as follows: 140 patients received systemic therapy and 73 received radiation therapy. A total of 113/140 (80.71%) patients undergoing systemic therapy were positive in the pre-screening of the NRS-2002; the risk of malnutrition was observed in 93/113 (66.43%) patients. A total of 56/73 (76.71%) of radiotherapy patients were positive in pre-screening, and 48/56 (65.75%) were also at risk of malnutrition after completing the NRS-2002. There were no significant differences in the rate of patients at risk between both groups.

### 3.2. Nutritional Deficiency—An Age-Independent Challenge

**Figure 1:**
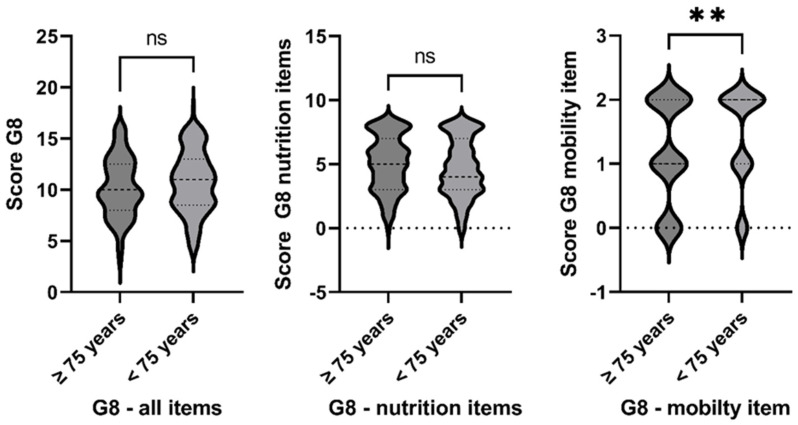
Nutrition deficiency is not dependent on patient age; however, mobility is lower in patients ≥75 years. ns—non significant, **—*p*-value < 0.01.

A total of 263/300 (87.67%) patients scored a value ≤ 14 points in the G8 and were therefore considered conspicuous. The mean G8 score was 10.55 points (+/− 2.97). Items of the G8 were categorized into the following groups: “nutrition”, “mobility”, and “others”. Overall, we observed no significant difference in the points scored between patients of the age cohort < 75 years compared to patients ≥ 75 years for the categories of nutrition and others. Younger (<75 years) patients, however, scored better on the mobility items (Figure 1).

### 3.3. Dietary Change and Cancer Diet

As part of the screening, we also interviewed three hundred patients using a nutrition module for diet changes. A total of 122/300 (40.67%) of the hospitalized patients stated that they were avoiding certain foods. Abstaining from alcohol was mentioned most frequently by 24/122 (19.67%) patients. This was closely followed by the avoidance of meat products and foods labeled as “solid/hard”, each with 23/122 (18.85%) mentions. After being diagnosed with cancer, 49/300 (16.33%) patients preferred certain foods. Most frequently listed were liquid and pureed food 22/49 (44.90%) or soft food 10/49 (20.41%). Patients were also asked about the intake of dietary supplements. Overall, 106/300 (35.33%) used dietary supplements. A cancer diet was denied by the majority (278/299, 92.98%) of patients. No significant difference between age cohorts or gender was observed concerning diet changes. Patients of the ≥ 85 years age cohort used nutritional supplements more often than younger patients. However, the sample size (13 patients ≥ 85 years) was very small. Subgroup analysis did not reveal differences between cancer entities except for lung cancer. Compared to other patients, lung cancer patients are significantly more prone to avoiding certain foods (*p* = 0.018). All subgroups are depicted in Table 3.

### 3.4. Symptom Burden—An Underestimated Player

**Figure 2:**
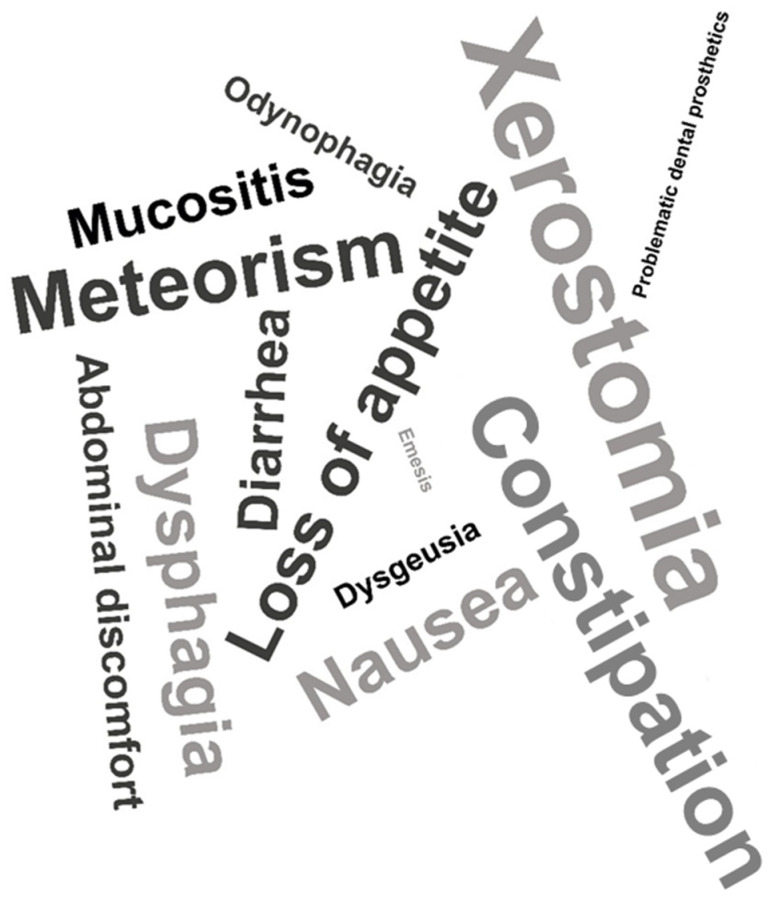
Symptom burden in cancer patients under therapy. Word cloud: size of words corresponds to the frequency of the symptom mentioned.

Side effects of cancer therapy may have an influence on nutritional status. To estimate symptom load, we added a simple symptom burden module to our survey. The frequency of single symptoms related to nutrition was as follows: xerostomia—178/300 (59.33%) patients, loss of appetite—122/300 (40.67%) patients, dysgeusia—93/300 (31.00%) patients, constipation—80/300 (26.67%) patients, meteorism—70/300 (23.33%) patients, nausea—64/300 (21.33%) patients, dysphagia—63/300 (21.00%), diarrhea—49/300 (16.33%) patients, oral mucositis—48/300 (16.00%) patients, abdominal discomfort—38/300 (12.67%) patients, odynophagia—30/300 (10.00%) patients, and emesis—17/300 (5.67%) patients (Figure 2).

Symptom burden has an influence on nutritional status and risk of malnutrition: an analysis of symptoms and malnutrition risk revealed that xerostomia in particular is significantly (*p* = 0.008) associated with being at risk of malnutrition (positive NRS-2002). Patients with dysgeusia showed at least a trend toward a risk of malnutrition (*p* = 0.100). Other symptoms showed no association with the risk of malnutrition. A detailed overview of subgroup analysis is depicted in Table 4.

Certain cancer entities are associated with a distinct symptom profile. Comparing patients with hematological neoplasms and solid tumors showed that dysphagia (*p* < 0.001), odynophagia (*p* = 0.010), and constipation (*p* = 0.032) occur significantly more frequently in patients with solid tumors. In contrast, patients with hematological diseases reported diarrhea significantly more frequently (*p* = 0.016). They also suffered significantly more frequently from nausea (*p* = 0.042) but not from emesis. There was a trend for patients with hematological neoplasms to report dysgeusia more frequently. Furthermore, patients reported an increased loss of appetite (*p* = 0.069, Table 5).

**Figure 3:**
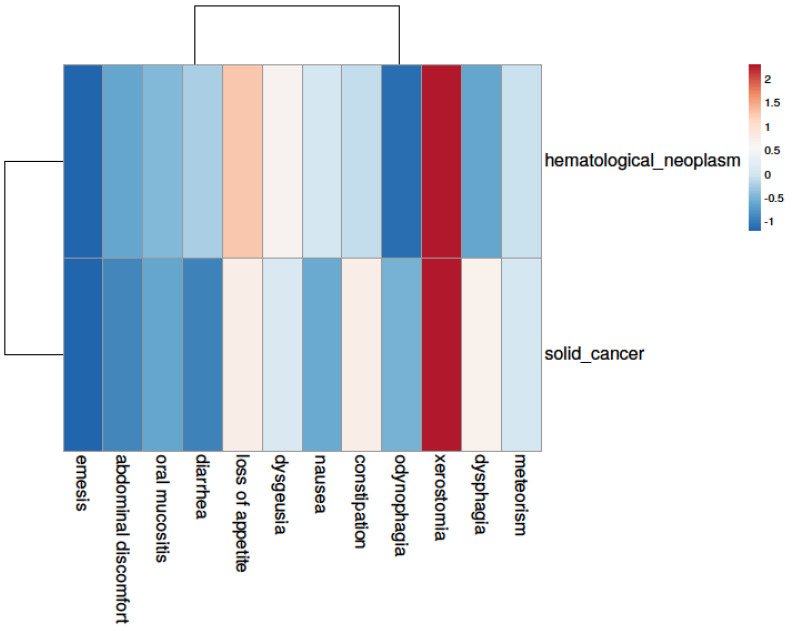
Patient groups show distinct symptom patterns. While xerostomia and meteorism are common problems in both groups, patients with hematological neoplasms are more prone to suffering from loss of appetite, dysgeusia, or nausea. Patients with solid tumors complain more often about constipation, dysphagia, and odynophagia.

Depicting symptom patterns by heat-map (Figure 3), we were able to observe certain symptom patterns that were more typical for patients with hematological neoplasms (e.g., loss of appetite, dysgeusia, nausea) or patients suffering from solid cancers (e.g., dysphagia, odynophagia, constipation).

A significant correlation was found between radiotherapy and dysphagia (*p* < 0.001) and odynophagia (*p* = 0.017). We observed a trend toward less frequent dysgeusia in patients who were recently diagnosed compared to cancer patients under prolonged therapy (*p* = 0.074). The symptom burden in relation to treatment modality is depicted in Table 6.

In regard to symptom burden, there was a significant difference between the age cohorts (<75 years; ≥75 years). The cohort of patients over 75 years was significantly more likely to have xerostomia (*p* = 0.015), dysphagia (*p* = 0.110), and constipation (*p* = 0.08). In contrast, dysgeusia (*p* = 0.120) and diarrhea (*p* = 0.003) were described more frequently in the cohort of younger patients (age < 75 years, also refer to Table 7).

## 4. Discussion

Older patients with cancer are a diverse group of people. Guidelines recommend considering biological and not chronological aging as well as health status when stratifying cancer patients for treatment [19]. The G8 screening tool is able to identify frail older patients. In addition to items on mobility, the neuropsychological situation, and medication intake, the screening primarily covers nutritional status (three question items, [13]). However, using the G8, we identified more than four out of five patients (87%) as frail, and patients mainly showed a decrease in points in nutrition items. This merits combining the G8 with established tools used for malnutrition screening. Recognizing the risk of malnutrition in older patients is important. Malnourished older patients are more prone to developing disease-associated complications, which in turn leads to longer hospitalization and poorer outcomes [20]. Accordingly, the European Society for Clinical Nutrition and Metabolism (ESPEN) recommends (1) routine and regular malnutrition screening for all patients diagnosed with cancer and argues for (2) repeated screening and more specific assessments for patients with a high-risk profile [4]. In 2016, the Global Leadership Initiative on Malnutrition (GLIM) established the “GLIM criteria”, a well-defined definition of malnutrition. These criteria comprise low BMI, non-volitional weight loss, reduced muscle mass, inflammation, disease burden, and decreased food intake [21]. The NRS-2002 covers a major part of these criteria and is also recommended by the German Society for Nutritional Medicine for screening inpatients [22]. Therefore, we combined the NRS-2002 with the G8 for our study.

While the German Society for Nutritional Medicine estimates that around half of all older people are at risk of malnutrition [23], our data demonstrates that more than two out of three older cancer patients are at risk. This higher risk of malnutrition fits the previous data of Shaw et al. [3], who described malnutrition in 71% of cancer patients. The risk of malnutrition is also higher in our cohort of older individuals compared to data from a previous study. While Döring et al. reported an overall risk of malnutrition of 37% in oncological patients and specifically described a risk of 44% in patients with hematological neoplasm [12], we observed a risk of 70% (all patients) and 74% (hematological neoplasm) respectively. As both studies used the NRS-2002 to assess the risk of malnutrition, this demonstrates that older people are an even more vulnerable patient group benefiting from nutritional screening.

Reflecting the risk of malnutrition in patients with solid tumors and hematological neoplasms, we observed a trend toward a higher risk of malnutrition in the latter. This is not surprising since different cancer entities are associated with an entity-specific risk, as each oncological treatment modality is characterized by a specific pattern of side effects (e.g., locoregional—radiation therapy/surgery, systemic—oral mucositis, [24,25]). Interestingly, we did not observe a significant difference in malnutrition risk rates when comparing patients with aggressive hematological neoplasms (aggressive lymphoma/acute leukemia) to patients with more chronic/indolent hematological disease (indolent lymphoma/chronic leukemia/MDS/MPN). The lower malnutrition risk rate in patients undergoing cellular immunotherapy compared to the overall population and patients with hematological neoplasms is probably due to a selection bias—only patients in good health with low comorbidities are considered for cellular immunotherapy [26].

Neither the aforementioned GLIM criteria [21] nor the NRS-2002 [10] take into account, that cancer patients may change their diet during oncological treatment. Cancer diets are a controversy [27,28,29], and oncologists are aware that cancer diets often diverge from official dietary guidelines such as the American Cancer Society or the American Institute for Cancer Research/World Cancer Research Fund [28]. Cancer diets are not only restricted to regimes offered by alternative medicine. During recent years, data showed that dietary restrictions prescribed by conventional medicine such as a neutropenic diet do not have a positive impact on infection rates in patients undergoing stem cell transplantation [30]. Fortunately, recent data from a German study showed that only a small minority of cancer patients follow a specific cancer diet [12], which fits the observation we made in our study. However, avoiding or preferring certain foods as well as using nutritional supplements may also influence nutritional status. To close this information gap, our group previously proposed a four-item questionnaire assessing cancer diet/dietary changes and the intake of micronutrients [14]. A pilot trial revealed that up to a third of cancer patients showed dietary changes after being diagnosed with cancer [12]. In this study, we observe that older patients are more prone to avoiding specific food (41%) compared to the younger cohort of Döring et al. (27%, [12]). Overall, abstaining from hard food or preferring soft/pureed nutrition seems to be a recurring topic. The latter could be interpreted as an adaption to the side effects of cancer therapy (e.g., no hard foods—oral mucositis, dysphagia, or odynophagia). These symptoms are often interconnected in patients undergoing radio(chemo)therapy. If a change of dietary habits is caused by therapy and thus influences nutritional status, we should (1) be aware of the side effects in order to (2) ameliorate supportive care and alleviate symptom burden. Further, a lower symptom burden also affects quality of life independent of weight loss [31], and supportive care provides patients with relief regardless of disease stage [32]. Following this rationale, we added a symptom burden questionnaire to our survey. In doing so, we aimed to identify the individual needs of patients, enabling oncological staff to ensure a patient-centered multimodal supportive therapy.

Surprisingly, patients’ chief complaints were xerostomia, loss of appetite, and dysgeusia. While this could be partly explained by treatment modality (radio(chemo)chemotherapy), treatment modality alone does not explain the high percentage of symptom burden. However, xerostomia is known to be more common in older people. Additionally, therapies such as radiation therapy but also chemo- or immunotherapy further decrease the salivation rate. Dysgeusia in turn often accompanies xerostomia, and sufficient salivation also aids digestion [33]. Digestion troubles of the upper gastrointestinal tract are not always a clear-cut symptom, and patients tend to describe the symptom as a “loss of appetite” or abdominal discomfort. Recognizing and addressing xerostomia is important, as we were able to observe a significant association between xerostomia and patients at risk of malnutrition. In addition to these symptoms, which are common in both patients with solid tumors or hematological neoplasm, we also observed specific symptom patterns in these groups. Some symptom associations are easily explained by the treatment modality: dysphagia or odynophagia are common side effects of radiation-induced esophagitis [25], and most of our patients with solid cancer (73/103) underwent radiotherapy.

Anticipating and (in the best case) preventing the side effects of oncological treatment is one of the core pillars of supportive care. An adapted, dietary, and symptom-based screening for malnutrition offers a chance to detect patients at risk before they develop cancer cachexia. As the screening proposed by our group also touches on the topics of diet and symptom burden, it could be also used as a standardized tool to enter a conversation about nutrition itself, educating patients and raising awareness. Understanding the diagnosis of “cancer” and the various phases of the disease as well as the necessity of a balanced diet can be of central importance [34,35]. The knowledge of symptoms and nutrition enables patients to become active themselves, thus empowering self-efficacy.

### Limitations

We conducted a cross-sectional study including 300 older inpatients with cancer (age ≥ 65 years). Our cohort was a mix of patients with solid cancer and hematological neoplasm. While subgroup analysis was possible for patients with hematological disease, the sample size was too small to compare different entities of solid cancers (except for lung cancer). Overall, subgroup analysis should be interpreted carefully due to the small sample size. While patients ≥ 85 years were included, we only surveyed 13 individuals, which is not representative. Here, a larger sample size is required to make assumptions on nutritional risk and symptom burden in this particular age group. No distinction between patients with curative or palliative concepts was made. We also have to consider that our study’s design only identified patients at risk but did not confirm manifesting malnutrition by nutritional assessment. The screening results were forwarded to the treating physicians. A shortcoming of this anonymous study is that we could not follow up on whether treating physicians initiated a deeper nutritional assessment of patients at risk or not. As the data analysis was mainly descriptive, we did not consider adjusting for multiple testing. Overall, our screening tool is reductive. Screening results should be used as a starting point for a deeper, more detailed assessment and the counseling of patients at risk. No information on comorbidities such as gastrointestinal diseases or other factors influencing nutrition (surgery, irradiated fields) was assessed.

## 5. Conclusions

**Figure 4:**
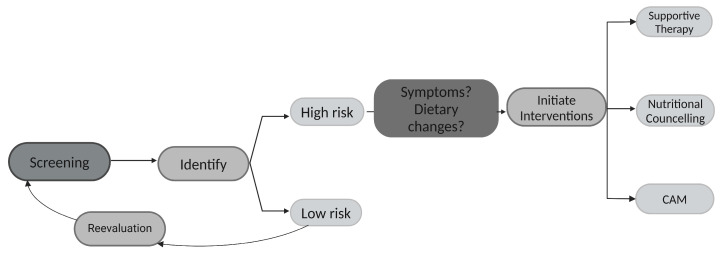
Flow chart: optimized screening for malnutrition in older cancer patients; CAM—complementary and alternative medicine.

Malnutrition is common in cancer patients, and older people are especially at risk. The reasons for developing malnutrition are diverse, and both systemic and local cancer therapy have an impact on patients’ nutritional status. Both the G8 and the NRS-2002 are good screening tools to identify patients at risk. However, they do not offer any information on possible underlying causes. By considering both diet changes and symptom burden, the treating oncological staff gains more insight into factors that may contribute to malnutrition and subsequently is able to address these specifically, e.g., by nutritional intervention or optimizing supportive care (Figure 4).

## Figures and Tables

**Table 1:** Clinical characteristics.

Total [N]		300
**Sex**	Female	136 (45.33%)
	Male	164 (54.67%)
**Age**	Age cohort 65–74 years	190 (63.33%)
	Age cohort 75–84 years	97 (32.33%)
	Age cohort ≥ 85 years	13 (4.33%)
**Entity**	**Malignant hematology**	187 (62.33%)
	Lymphoma (aggressive)	45 (15.00%)
	Lymphoma (indolent)	67 (22.33%)
	Acute leukemia	39 (13.00%)
	Chronic leukemia	6 (2.00%)
	Myeloproliferative neoplasia	6 (2.00%)
	Myelodysplastic syndrome	7 (2.33%)
	**Solid Tumor**	113 (37.67%)
	Lung cancer (NSCLC)	31 (10.33%)
	Lung cancer (SCLC)	12 (4.00%)
	Breast cancer	2 (0.67%)
	Sarcoma	9 (3.00%)
	Lower gastrointestinal tract tumor	6 (2.00%)
	Other gynecological cancers	7 (2.33%)
	Urooncological cancer	10 (3.33%)
	Other	54 (18.00%)
**Treatment modality [N]**	**213**	
	Systemic treatment	140 (65.73%)
	Radiation therapy	73 (34.27%)
**Cellular immunotherapy**	Allogenic, autologous stem cell therapy + CAR-T-cell therapy	25/300 (8.33%)

CAR-T-cell therapy—chimeric antigen receptor-T-cell therapy; NSCLC—non-small cell lung cancer; SCLC—small-cell lung cancer.

**Table 2:** Analysis of the NRS-2002 screening results by cancer entity.

Entity	Total	Pre-Screen NRS-2002 Positive	NRS-2002 Positive	*p*-Value
	[N]	[N]	[N]	
Hematologocal neoplasm	187	153	138	0.1044
Solid tumors	113	86	71	
Lymphoma (aggressive)/acute leukemia	84	67	62	0.7638
Lymphoma (indolent)/chronic leukemia/MDS/MPN	84	68	61	
Lymphoma (aggressive)/acute leukemia	84	67	62	0.1917
Others	216	172	147	
Lung (NSCLC/SCLC)	44	34	27	0.1519
Others	256	205	182	
NSCLC	31	23	20	1.0000
Others	269	216	189	
SCLC	13	11	7	**0.0357**
Others	287	228	202	
Cellular immunotherapy	25	22	16	**0.0432**
Others	272	214	190	
ED	43	36	29	0.1816
Others	255	201	178	

NRS-2002—Nutritional Risk Score-2002, MDS—myelodysplastic syndrome, MPN—myeloproliferative neoplasia, NSCLC—non-small cell lung cancer, SCLC—small-cell lung cancer.

**Table 3:** Dietary changes sub-group analysis of age cohorts, sex, and cancer entity.

Age Cohort	Avoid Specific Food Yes	Avoid Specific Food No	*p*-Value
≥65–74 years	83	107	0.2540
75–84 years	35	62	
75–84 years	35	62	1.0000
≥85 years	4	9	
≥65–74 years	83	107	0.4032
≥85 years	4	9	
**Age cohort**	**Prefer specific food** **Yes**	**Prefer specific food** **No**	***p*-value**
≥65–74 years	33	157	1.0000
75–84 years	16	81	
75–84 years	16	81	0.2084
≥85 years	0	13	
≥65–74 years	33	157	0.1331
≥85 years	0	13	
**Age Cohort**	**Use of Additional Supplements** **Yes**	**Use of Additional Supplements** **No**	***p*-Value**
≥65–74 years	65	125	0.7916
75–84 years	31	66	
75–84 years	31	66	**0.0040**
≥85 years	10	3	
≥65–74 years	65	125	**0.0050**
≥85 years	10	3	
**Age Cohorts**	**Cancer Diet** **Yes**	**Cancer Diet** **No**	***p*-Value**
≥65–74 years	14	175	**0.3179**
75–84 years	4	93	
75–84 years	4	93	0.0346
≥85 years	3	10	
≥65–74 years	14	175	0.0836
≥85 years	3	10	
**Sex**	**Avoid Specific Food** **Yes**	**Avoid Specific Food** **No**	***p*-Value**
m	72	93	0.2878
f	50	85	
	**Prefer Specific Food** **Yes**	**Prefer Specific Food ** **No**	***p*-Value**
m	24	141	0.4328
f	25	110	
	**Use of Additional Supplements** **Yes**	**Use of Additional Supplements** **No**	***p*-Value**
m	59	106	0.1904
f	47	88	
	**Cancer Diet** **Yes**	**Cancer Diet** **No**	***p*-Value**
m	10	154	0.5046
f	11	124	
**Cancer Entity**	**Avoid Specific Food** **Yes**	**Avoid Specific Food** **No**	***p*-Value**
Lymphoma (aggressive)/acute leukemia	34	50	1.0000
Others	87	128	
Lymphoma (indolent)/chronic leukemia/MDS/MPN	32	52	0.6943
Others	89	126	
Lung cancer (NSCLC/SCLC)	10	33	**0.0179**
Others	111	145	
	**Prefer Specific Food** **Yes**	**Prefer Specific Food ** **No**	***p*-Value**
Lymphoma (aggressive)/acute leukemia	10	74	0.2257
Others	39	176	
Lymphoma (indolent)/chronic leukemia/MDS/MPN	12	72	0.6051
Others	37	178	
Lung cancer (NSCLC/SCLC)	5	38	0.5043
Others	44	212	
	**Use of Additional Supplements** **Yes**	**Use of Additional Supplements** **No**	***p*-Value**
Lymphoma (aggressive)/acute leukemia	25	59	0.2270
Others	81	134	
Lymphoma (indolent)/chronic leukemia/MDS/MPN	31	53	0.7884
Others	75	140	
Lung cancer (NSCLC/SCLC)	15	28	1.0000
Others	91	165	
	**Cancer Diet** **Yes**	**Cancer Diet** **No**	***p*-Value**
Lymphoma (aggressive)/acute leukemia	6	78	0.8022
Others	14	200	
Lymphoma (indolent)/chronic leukemia/MDS/MPN	4	80	0.6069
Others	16	198	
Lung cancer (NSCLC/SCLC)	1	42	0.3277
Others	19	236	

MDS—myelodysplastic syndrome, MPN—myeloproliferative neoplasia, NSCLC—non-small cell lung cancer, SCLC—small-cell lung cancer.

**Table 4:** Symptom burden in relation to nutritional status.

Nutritional Status	Symptom Positive [N]	Symptom Negative [N]	*p*-Value
	**Mucositis**		
**NRS-2002 positive**	39	170	1.000
**NRS-2002 negative**	5	25	
	**Dysgeusia**		
**NRS-2002 positive**	76	133	0.0997
**NRS-2002 negative**	7	23	
	**Dysphagia**		
**NRS-2002 positive**	50	159	0.8183
**NRS-2002 negative**	6	24	
	**Diarrhea**		
**NRS-2002 positive**	34	175	1.0000
**NRS-2002 negative**	5	25	
	**Meteorism**		
**NRS-2002 positive**	51	157	0.6554
**NRS-2002 negative**	6	24	
	**Nausea**		
**NRS-2002 positive**	47	162	0.6441
**NRS-2002 negative**	8	22	
	**Xerostomia**		
**NRS-2002 positive**	138	71	**0.0082**
**NRS-2002 negative**	12	18	
	**Loss of appetite**		
**NRS-2002 positive**	104	105	0.3362
**NRS-2002 negative**	12	18	
	**Odynophagia**		
**NRS-2002 positive**	23	186	1.0000
**NRS-2002 negative**	3	27	
	**Constipation**		
**NRS-2002 positive**	64	144	0.2863
**NRS-2002 negative**	6	24	
	**Abdominal discomfort**		
**NRS-2002 positive**	31	178	0.3929
**NRS-2002 negative**	2	28	
	**Emesis**		
**NRS-2002 positive**	14	195	1.0000
**NRS-2002 negative**	2	28	

Nutritional Risk Score-2002—NRS-2002.

**Table 5:** Symptom burden comparing patients with either hematological neoplasms or solid tumors.

Cancer Entity	Symptom Positive [N]	Symptom Negative [N]	*p*-Value
	**Mucositis**		
**Hematological neoplasm**	32	155	0.5212
**Solid tumor**	16	97	
	**Dysgeusia**		
**Hematological neoplasm**	65	122	0.0730
**Solid tumor**	28	85	
	**Dysphagia**		
**Hematological neoplasm**	26	161	**0.0002**
**Solid tumor**	37	76	
	**Diarrhea**		
**Hematological neoplasm**	38	149	**0.0161**
**Solid tumor**	11	102	
	**Meteorism**		
**Hematological neoplasm**	43	143	0.8887
**Solid tumor**	27	86	
	**Nausea**		
**Hematological neoplasm**	47	140	**0.0423**
**Solid tumor**	17	96	
	**Xerostomia**		
**Hematological neoplasm**	115	71	0.3317
**Solid tumor**	63	50	
	**Loss of appetite**		
**Hematological neoplasm**	84	103	0.0686
**Solid tumor**	38	75	
	**Odynophagia**		
**Hematological neoplasm**	12	175	**0.0098**
**Solid tumor**	18	95	
	**Constipation**		
**Hematological neoplasm**	42	145	**0.0319**
**Solid tumor**	38	74	
	**Abdominal discomfort**		
**Hematological neoplasm**	26	161	0.4759
**Solid tumor**	12	101	
	**Emesis**		
**Hematological neoplasm**	10	177	0.7995
**Solid tumor**	7	106	

**Table 6:** Symptom burden in relation to treatment modality.

Treatment Modality	Symptom Positive [N]	Symptom Negative [N]	*p*-Value
	**Mucositis**		
**Systemic treatment**	23	117	0.8481
**Radiation therapy**	13	60	
	**Dysgeusia**		
**Systemic treatment**	49	91	0.2836
**Radiation therapy**	20	53	
	**Dysphagia**		
**Systemic treatment**	19	121	**0.0002**
**Radiation therapy**	27	46	
	**Diarrhea**		
**Systemic treatment**	20	120	0.3904
**Radiation therapy**	7	66	
	**Meteorism**		
**Systemic treatment**	29	110	0.7271
**Radiation therapy**	17	56	
	**Nausea**		
**Systemic treatment**	25	115	0.7018
**Radiation therapy**	11	62	
	**Xerostomia**		
**Systemic treatment**	83	56	0.6611
**Radiation therapy**	41	32	
	**Loss of appetite**		
**Systemic treatment**	55	85	0.2296
**Radiation therapy**	22	51	
	**Odynophagia**		
**Systemic treatment**	12	128	**0.0168**
**Radiation therapy**	15	58	
	**Constipation**		
**Systemic treatment**	37	102	1.0000
**Radiation therapy**	20	53	
	**Abdominal discomfort**		
**Systemic treatment**	16	124	0.8176
**Radiation therapy**	7	66	
	**Emesis**		
**Systemic treatment**	6	134	0.7387
**Radiation therapy**	4	69	

**Table 7:** Symptom burden in relation to age cohorts.

Age Cohort	Symptom Positive [N]	Symptom Negative [N]	*p*-Value
	**Oral Mucositis**		
**<75 a**	29	161	0.744
**≥75 a**	19	91	
	**Dysgeusia**		
**<75 a**	65	125	0.1219
**≥75 a**	28	82	
	**Dysphagia**		
**<75 a**	34	156	0.1052
**≥75 a**	29	81	
	**Diarrhea**		
**<75 a**	40	150	**0.0034**
**≥75 a**	9	101	
	**Meteorism**		
**<75 a**	44	145	1.0000
**≥75 a**	26	84	
	**Nausea**		
**<75 a**	43	147	0.5590
**≥75 a**	21	89	
	**Xerostomia**		
**<75 a**	103	87	**0.0146**
**≥75 a**	75	34	
	**Loss of appetite**		
**<75 a**	75	115	0.6262
**≥75 a**	47	63	
	**Odynophagia**		
**<75 a**	17	173	0.4308
**≥75 a**	13	97	
	**Constipation**		
**<75 a**	44	145	0.0799
**≥75 a**	36	74	
	**Abdominal discomfort**		
**<75 a**	27	163	0.3684
**≥75 a**	11	99	
	**Emesis**		
**<75 a**	11	179	1.0000
**≥75 a**	6	104	

## Data Availability

The data presented in this study are available on request from the corresponding author.

## Appendix A

Symptom-based screening for malnutrition, combining G8, NRS-2002, and the authors’ four-item questionnaire (German and English).

**Geriatrisches Screening und Symptomorientierte Ernährungsanamnese**					
**Alter (Jahre)**	65–74		75–84		>85
**Geschlecht**	Männl.		Weibl.		Divers

**Entität (Neoplasie)**	
Hämatologisch	Solider Tumor
Lymphom (aggressiv)	Lunge (NSCLC)
Lymphom (indolent)	Lunge (SCLC)
Akute Leukämie	Brustkrebs
Chronische Leukämie	Sarkom
Myeloproliferative Syndrome	HNO-Tumor
Myelodysplastische Syndome	Hodentumor
Andere______________________	Andere_____________________

**Anthropometrisc**	**he Daten**	
Größe [m]	Gewicht [kg]	BMI [kg/m^2^]
Handkraft		

**Symptomorientier Ernährungsanamnese**	
Orale Mukositis	Xerostomie
Geschmacksveränderung	Appetitlösigkeit
Dysphagie	Odynophagie
Diarrhoe	Obstipation
Blähung	Völlegefühl
Übelkeit	Erbrechen
Sonstige	
Zahnersatz/Prothese	
PEG-Sonde	
Andere Besonderheiten: ________________	

**G8-Screening-Test****(Deutscher Bogen Übernommen aus HONECKER 2015)**				
	Fragen	Score		Mögliche Antworten
A	Hat die Nahrungsaufnahme in den letzten 3 Monaten aufgrund von Appetitverlust, Verdauungsproblemen, Kau-oder Schluckproblemen abgenommen?	0: 1: 2:	schwere Einschränkungen mäßige Einschränkung der Nahrungszufuhr Normale Nahrungsaufnahme	
B	Gewichtsverlust in den letzten 3 Monaten?	0: 1:	Gewichtsverlust > 3 kg unbekannt	
		2:	Gewichtsverlut zwischen 1 und 3 kg	
		3:	Kein Gewichtsverlust	
C	Mobilität?	0:	Bett oder Stuhl	
		1:	kann aus Bett oder Stuhl aufstehen, aber nicht nach draußen	
		2:	Geht nach draußen	
E	Neuropsychologische Probleme?	0:	schwere Demenz oder Depression	
		1:	Milde Demenz oder Depression	
		2:	keine psychologischen Probleme	
F	Body-Mass-Index	0:	BM < 19	
		1:	BMI 19–21	
		2:	21 bis < 23	
		3	BMI > 23	
H	Nimmt mehr als 3 Medikamente am Tag ein	1: 0:	Nein. Ja.	
P	Verglichen mit Gleichaltrigen, wie schätzt der Patient seinen Zustand ein?	0: 0.5: 1: 2:	Nicht so gut. Weiß nicht. Gleich gut. Besser.	
	Alter	0:	>85	
		1:	80–85	
		2:	<80	
Total Score (0–17)				
Total Score: Gesamtsumme der erreichten Punkte; cut-off: ≤14 Punkte = auffälliges Screening				

NRS2002 (nach nach Kondrup J et al., Clinical Nutrition 2003; 22: 415–421 [10])					
Vorscreening				Ja	Nein
**Ist der Body Mass Index < 20.5 kg/m^2^?**					
**Hat der Patient die letzten 3 Monate an Gewicht verloren**					
**War die Nahrungszufuhr in der letzten Woche vermindert?**					
**Ist der Patient schwer erkrankt (z. B. Intensivtherapie)?**					
Screening					
**Störung des Ernährungszustandes**	Punkte	+	Krankheitsschwere	Punkte	
**Keine**	0		Keine	0	
**Mild** **Gewichtsverlust > 5% in 3** **Monaten oder Nahrungszufuhr** **<50–75% des Bedarfs in der** **Vorwoche**	1		Mild Schenkelhalsfraktur, chronische Erkrankung mit Komplikationen, Krebsleide	1	
**Mäßig** **Gewichtsverlust > 5% in 2 Monaten oder BMI 18.5–20.5 kg/m^2^ und reduzierter Allgemeinzustand oder** **Nahrungszufuhr 20–50% des** **Bedarfs in der Vorwoche**	2		Mild Große Bauchchirurgie, Schlaganfall, Pneumonie, hämatologische Krebserkrankung	2	
**Schwer** **Gewichtsverlust > 5% in 1 Monat oder BMI < 18.5 kg/m^2^ und reduzierter Allgemeinzustand oder Nahrungszufuhr 0–25% des** **Bedarfs in der Vorwoche**	3		Schwer Kopfverletzung, Knochenmark- transplantation, Intensivpflichtige Patienten	3	
**+1 Punkt, wenn Alter** ≥ **70 Jahre**					
≥*3 Punkte*	***Risiko für Malnutrition liegt vor, Erstellung eines Ernährungsplans***				
<*3 Punkte*	***wöchentlich Screening wiederholen***				
Originalpublikation: Kondrup et al. 2003, Nutritional risk screening (NRS 2002): a new methodbased on an analysis of controlledclinical trials; Clinical Nutrition (2003) 22(3): 321–336 [10]					

Screening Ernährungsumstellung/Krebsdiät (nach Büntzel und Büntzel 2022 [14])			
Haben Sie Ihre Ernährungsgewohnheiten verändert, seitdem Sie von Ihrer Krebsdiagnose wissen?			
Ja			***Nein***
Verzichten oder vermeiden Sie bestimme Nahrungsmittel?	*JA, _______________*	*NEIN*	*Bevor Sie mit einer* *Krebsdiöt beginnen, sprechen Sie bitte mit Ihrer/m HausärztIn oder behandelnde/n* *OnkologIn*
Bevorzugen Sie bestimmte Nahrungsmittel?	*JA, _______________*	*NEIN*	
Nehmen Sie Nahrungsergänzungsmittel ein?	*JA, _______________*	*NEIN*	
Folgen Sie besonderen Ernährungshinweise/Diätplänen?	*JA, _______________*	*NEIN*	

**Geriatric Screening and Nutritional Anamnesis**			
**Age (years)**	65–74	75–84	>85
**Sex**	Male	Female	Divers

**Cancer Entity**	
Hematological neoplasm	Solid cancer
Lymphoma (aggressive)	Lung cancer (NSCLC)
Lymphoma (non-aggressive)	Lung cancer (SCLC)
Acute leukemia	Breast cancer
Chronic leukemia	Sarcoma
Myeloproliferative syndrome	Head-neck cancer
Myelodysplastic syndrome	Testicular cancer
Others_______________	Others_______________

**Anthropometric Data**		
Height [m]	Weight [kg]	BMI [kg/m^2^]
Hand grip strength		

**Symptom Burden**	
Oral mucositis	Xerostomia
Dysgeusia	Loss of appetite
Dysphagia	Odynophagia
Diarrhea	Constipation
Bloating	Fullness
Nausea	Emesis
Others	
Dental prothesis	
Percutaneous endoscopic gastrostomy	
Other particularities: ________________	

**G8 Screening**				
	Question	Possible answers		Score
A	Has food intake declined over the past 3 months due to loss of appetite, digestive problems, chewing, or swallowing difficulties?	0: 1: 2:	Severe decrease Moderate decrease Normal food intake	
B	Weight loss during the last 3 months?	0: 1:	Weight loss > 3 kg unknown	
		2:	Weight loss between 1–3 kg	
		3:	No weight loss	
C	Mobility?	0:	Bed or chair bound	
		1:	Able to get out of bed/chair, but does not go out	
		2:	Goes out	
E	Neuropsychological problems?	0:	Severe dementia or depression	
		1:	mild dementia or depression	
		2:	No psychological problems	
F	Body-Mass-Index	0:	<19	
		1:	19–21	
		2:	21–≤23	
		3	>23	
H	More than three prescription drugs per day?	1: 0:	No. Yes.	
P	In comparison with other people of the same age, how does the patient consider his/her health status?	0: 0.5: 1: 2:	Not as good. Does not know. As good. Better.	
	Age	0:	>85 years	
		1:	80–85 years	
		2:	<80 years	
Total Score (0–17)				
Total Score: Sum of all points reached, cut-off: ≤14 points indicating a positive screening				

**NRS2002 (According to Kondrup J et al., Clinical Nutrition 2003; 22: 415–421 [10])**					
pre-screening				Yes	No
Is BMI ≤ 20.5?					
Has the patient lost weight within the last 3 months?					
Has the patient had a reduced dietary intake in the last week?					
Is the patient severely ill ? (e.g., in intensive therapy)					
**Screening**					
**Impaired nutritional status**	**Points**	**+**	**Severity of disease**	**Points**	
Normal nutritional status	0		Keine	0	
Mild Wt loss > 5% in 3 mths or Food intake below 50–75% of normal requirement in preceding week	1		Mild Hip fracture Chronic patients, in particular with acute complications, chronic hemodialysis, diabetes, oncology	1	
Moderate Wt loss > 5% in 2 mths or BMI 18.5–20.5 + impaired general condition or Food intake 25–50% of normal requirement in preceding week	2		Moderate Major abdominal surgery, stroke, severe pneumonia, hematologic malignancy	2	
Severe Wt loss 45% in 1 mth (>15% in 3 mths) or BMI < 18.5 + impaired general condition or Food intake 0–25% of normal requirement in preceding week in preceding week.	3		Severe Head injury, bone marrow transplantation, intensive care patients	3	
if ≥70 years: add 1 to total score above = age-adjusted total score					
≥*3 points*	the patient is nutritionally at-risk and a nutritional care plan is initiated				
<*3 points*	weekly rescreening of the patient. If the patient, e.g., is scheduled for a major operation, a preventive nutritional care plan is considered to avoid the associated risk status.				
Kondrup et al. 2003, Nutritional risk screening (NRS 2002): a new method based on an analysis of controlled clinical trials; Clinical Nutrition (2003) 22(3): 321–336 [10]					

**Screening Dietary Changes (Büntzel and Büntzel 2022 [14])**			
Did you change your dietary habits after being diagnosed with cancer?			
Yes			***No***
Do you avoid certain foods?	*YES, _______________*	*No*	*Before starting a cancer diet, please seek advice from your family doctor or treating oncologist.*
Do you prefer certain foods?	*YES, _______________*	*No*	
Do you use nutritional supplements?	*YES, _______________*	*No*	
Do you follow a specific cancer diet?	*YES, _______________*	*No*	

## Appendix B

STROBE Statement—checklist of items that should be included in reports of observational studies adapted from [15], 2007 Vandenbroucke et al., Creative Commons Attribution License.

	**Item No**	**Recommendation**	**Page No**
**Title and abstract**	1	(*a*) Indicate the study’s design with a commonly used term in the title or the abstract	1
		(*b*) Provide in the abstract an informative and balanced summary of what was done and what was found	1
**Introduction**			
Background/rationale	2	Explain the scientific background and rationale for the investigation being reported	1f
Objectives	3	State specific objectives, including any prespecified hypotheses	2
**Methods**			
Study design	4	Present key elements of study design early in the paper	2ff
Setting	5	Describe the setting, locations, and relevant dates, including periods of recruitment, exposure, follow-up, and data collection	2ff
Participants	6	*Cross-sectional study*—Give the eligibility criteria and the sources and methods of selection of participants	2
Variables	7	Clearly define all outcomes, exposures, predictors, potential confounders, and effect modifiers. Give diagnostic criteria, if applicable	2ff
Data sources/measurement	8 *	For each variable of interest, give sources of data and details of methods of assessment (measurement). Describe comparability of assessment methods if there is more than one group	2ff
Bias	9	Describe any efforts to address potential sources of bias	2
Study size	10	Explain how the study size was arrived at	2
Quantitative variables	11	Explain how quantitative variables were handled in the analyses. If applicable, describe which groupings were chosen and why	2ff
Statistical methods	12	(*a*) Describe all statistical methods, including those used to control for confounding	3
		(*b*) Describe any methods used to examine subgroups and interactions	3
		(*c*) Explain how missing data were addressed	3
		*Cross-sectional study*—If applicable, describe analytical methods taking account of sampling strategy	n. a.
		(*e*) Describe any sensitivity analyses	n. a.
**Results**				
Participants	13 *	(a) Report numbers of individuals at each stage of study—e.g., numbers potentially eligible, examined for eligibility, confirmed eligible, included in the study, completing follow-up, and analyzed	3ff	
		(b) Give reasons for non-participation at each stage	n. a.	
		(c) Consider use of a flow diagram	n. a.	
Descriptive data	14 *	(a) Give characteristics of study participants (e.g., demographic, clinical, social) and information on exposures and potential confounders	3ff	
		(b) Indicate number of participants with missing data for each variable of interest	5	
Outcome data	15 *	*Cohort study*—Report numbers of outcome events or summary measures over time	n. a.	
		*Case-control study*—Report numbers in each exposure category, or summary measures of exposure	n. a.	
		*Cross-sectional study*—Report numbers of outcome events or summary measures	3ff	
Main results	16	(*a*) Give unadjusted estimates and, if applicable, confounder-adjusted estimates and their precision (e.g., 95% confidence interval). Make clear which confounders were adjusted for and why they were included	3ff	
		(*b*) Report category boundaries when continuous variables were categorized	3ff	
		(*c*) If relevant, consider translating estimates of relative risk into absolute risk for a meaningful time period	n. a.	
Other analyses	17	Report other analyses conducted—e.g., analyses of subgroups and interactions, and sensitivity analyses	3ff	
**Discussion**				
Key results	18	Summarize key results with reference to study objectives	17ff	
Limitations	19	Discuss limitations of the study, taking into account sources of potential bias or imprecision. Discuss both direction and magnitude of any potential bias	19f	
Interpretation	20	Give a cautious overall interpretation of results considering objectives, limitations, multiplicity of analyses, results from similar studies, and other relevant evidence	19f	
Generalizability	21	Discuss the generalizability (external validity) of the study results	17ff	
**Other information**				
Funding	22	Give the source of funding and the role of the funders for the present study and, if applicable, for the original study on which the present article is based	20	

* Give information separately for cases and controls in case-control studies and, if applicable, for exposed and unexposed groups in cohort and cross-sectional studies.
